# Fine-scale mapping of meiotic recombination in Asians

**DOI:** 10.1186/1471-2156-14-19

**Published:** 2013-03-08

**Authors:** Thomas Bleazard, Young Seok Ju, Joohon Sung, Jeong-Sun Seo

**Affiliations:** 1 Genomic Medicine Institute (GMI), Medical Research Center, Seoul National University, Seoul, South Korea; 2 College of Natural Sciences, Seoul National University, Seoul, South Korea; 3 Department of Epidemiology, School of Public Health and Institute of Environment and Health, Seoul National University, Seoul, South Korea; 4 Department of Biochemistry and Molecular Biology, Seoul National University College of Medicine, Seoul, South Korea

**Keywords:** Recombination, Hotspot, Asian, Genetic map

## Abstract

**Background:**

Meiotic recombination causes a shuffling of homologous chromosomes as they are passed from parents to children. Finding the genomic locations where these crossovers occur is important for genetic association studies, understanding population genetic variation, and predicting disease-causing structural rearrangements. There have been several reports that recombination hotspot usage differs between human populations. But while fine-scale genetic maps exist for European and African populations, none have been constructed for Asians.

**Results:**

Here we present the first Asian genetic map with resolution high enough to reveal hotspot usage. We constructed this map by applying a hidden Markov model to genotype data for over 500,000 single nucleotide polymorphism markers from Korean and Mongolian pedigrees which include 980 meioses. We identified 32,922 crossovers with a precision rate of 99%, 97% sensitivity, and a median resolution of 105,949 bp. For direct comparison of genetic maps between ethnic groups, we also constructed a map for CEPH families using identical methods. We found high levels of concordance with known hotspots, with approximately 72% of recombination occurring in these regions. We investigated the hypothesized contribution of recombination problems to age-related aneuploidy. Our large sample size allowed us to detect a weak but significant negative effect of maternal age on recombination rate.

**Conclusions:**

We have constructed the first fine-scale Asian genetic map. This fills an important gap in the understanding of recombination pattern variation and will be a valuable resource for future research in population genetics. Our map will improve the accuracy of linkage studies and inform the design of genome-wide association studies in the Asian population.

## Background

Homologous recombination during meiosis results from the resolution of programmed DNA double-strand breaks by crossover between non-sister chromatids. Recombination is concentrated into hotspots roughly 2 kb in length scattered throughout the genome [1]. Recombination rates between markers are used to construct genetic maps. These are important for linkage studies and also genome-wide association studies, since tagging single nucleotide polymorphisms (SNPs) depend on linkage-disequilibrium structures. Early human genetic maps were constructed using sparse microsatellite markers in Icelandic and CEPH (Center d’Etude du Polymorphisme Humain) families [2-4]. More recent studies have used SNP markers to reveal the locations of recombination with much greater resolution in Hutterites and other European-American cohorts (Framingham Heart Cohort Study and Autism Genetic Resource Exchange) [5,6] and African-Americans [7]. These studies used algorithms based on the ancestral origin of alleles [7], and parent-sibling tracing approaches [5,6,8]. In contrast to family methods which can directly observe recombination, coalescent methods use population genetic data to estimate the genetic map as a parameter of a probabilistic model. This captures the recombination that shuffled genomes through the generations leading to the current population. Applying these methods to HapMap data achieved very high resolution and allowed the identification of hotspots using likelihood ratio tests [1,9].

For all applications, it is important for a genetic map to correspond well to the group to which it is applied. For example, the use of a map which accurately reflects the average recombination rate of a population will increase the power of gene identification studies. Some evidence suggests, however, that recombination patterns differ between populations. It is known that the DNA-binding protein PRDM9 plays a key role in specifying recombination sites [10]. The binding motif that PRDM9 recognises must change rapidly under a constant Red Queen dynamic because of the ‘hotspot paradox’ [11-13]. Studies of human populations of African and European descent have shown variation in alleles of *PRDM9* and local patterns of recombination [6,7]. Three minor alleles, in addition to the more common A and B haplotypes, were previously identified in Han Chinese coding for either 12 or 13 zinc fingers [14]. Interestingly, our preliminary genetic data from the Mongolian population suggest a larger diversity of *PRDM9* alleles, including novel SNPs predicted to affect DNA binding (Additional file 1). Given this, the genetic map of Asians may be different to those from other populations. Furthermore, the hotspots found by coalescent models receding many generations into the past may not be in use today.

In a previous work, we constructed a map for Mongolians using 1,039 microsatellite markers [15]. However, the resolution of the map was not sufficient to identify hotspot usage. There are currently no other Asian genetic maps available. Here we present a new genetic map, constructed using dense SNP markers in Mongolian and Korean pedigrees. The resolution of this map is now sufficient to reveal fine-scale patterns of recombination and Asian hotspot characteristics for the first time.

## Results and discussion

### Genetic map

**Figure 1:**
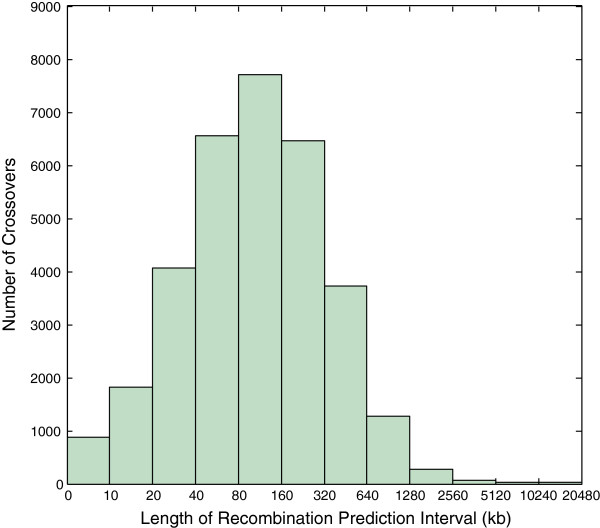
**Recombination prediction interval length distribution.** Histogram of the resolutions of recombination prediction intervals in all Asian meioses. Prediction intervals are determined non-probabilistically by our algorithm as the maximal region in which a state change may have occurred. The x-axis is scaled logarithmically.

We developed a novel algorithm to detect crossovers using family genotype data. This algorithm was based on a hidden Markov model as described in Methods. We tested the accuracy of our algorithm by applying it to simulated data with known crossover positions (see Additional file 1). This showed the sensitivity to be 97% and the precision rate to be 99%, which compares very well with previously published methods [16]. We used our algorithm to investigate recombination in 169 Mongolian and Korean nuclear families (Table 1). By inputting genotype data for these families, we were able to deduce recombination events in a total of 980 meioses. We applied consistency checks to the raw output, and removed from analysis 18 meioses which failed our criteria. Our final data-set contained 32,922 crossovers with a median resolution of 105,949 bp (Figure 1). The complete list of these recombination events (Additional file 2) and sex-averaged genetic maps for each chromosome (Additional file 3) are available for download as a resource for future research. Our data is comprehensive, including maternal recombinations on the X chromosome that have previously been neglected [5,6,17]. These reveal an irregular pattern of peaks and troughs, with X chromosome recombination largely clustered near the telomeres, at an average distance of 28 Mb from the two ends. Thus, by detecting the locations of recombination in Mongolian and Korean pedigrees, we have constructed a high-quality, fine-scale Asian genetic map.

**Table 1:** Pedigree structures

**Size of family (children)**	**Mongolian**	**Korean**	**CEPH families**
2	26	50	0
3	12	41	0
4	6	17	0
5	3	12	1
6	0	2	1
7	0	0	1
8	0	0	3
9	0	0	1
10	0	0	3
11	0	0	1
12	0	0	1
13	0	0	1
Total number of families	47	122	13
Total number of children	127	361	117

The composition of the Mongolian, Korean and CEPH family pedigrees, with the number of families of each size. Only families with at least two children are shown, since this is required for application of our algorithm. For analysis of maternal age effects, only families with at least three children were used.

### Ethnic comparison

**Figure 2:**
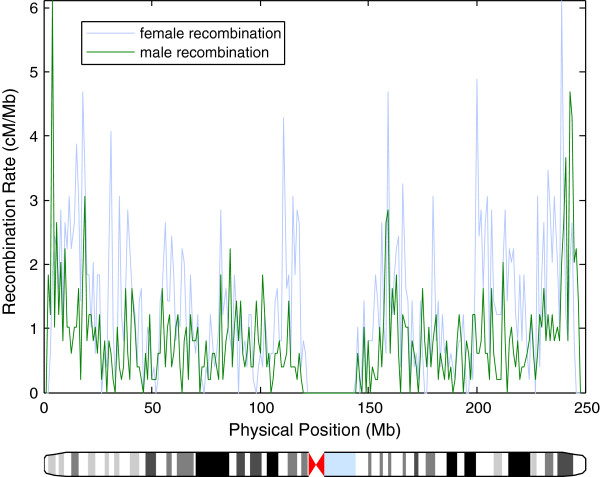
**Chromosome 1 genetic map.** Recombination rates across chromosome 1, smoothed to 1 Mb. Recombination events are plotted at the midpoint of their prediction interval, and events whose resolution is worse than 5 Mb are excluded. The chromosome ideogram is marked with cytogenetic G-banding data provided by the MATLAB package.

At the level of chromosomes, rate patterns are as expected with deserts at centromeres and spikes corresponding to increased paternal recombination near the end of chromosome arms as previously reported [3] (Figure 2). Asian males had an average of 26.3 (sample standard deviation = 3.3; 25.8-26.8 95% CI) and females had an average of 41.1 (s = 6.3; 40.1-42.0 95% CI) crossovers per meiosis (Table 2). These rates are comparable to previous studies of other populations [5,6,17]. In order to make a more direct comparison, we further applied our hidden Markov model to genotype data from CEPH families. A detailed comparison of the features of this map and the Mongolian and Korean maps is given in Table 3. Though we reported small but significant ethnic differences in genetic map length in our previous study [15], this direct comparison showed similar map lengths between Asians and CEPH families. We believe that the results of this study may be more accurate for several reasons as follows. First, this study made a direct comparison with identical software parameters and data handling. Second, cutting pedigrees into family quartets removes bias caused by different cohort family structures. For example, heuristic methods that consider all offspring together have been found to suffer downward bias in smaller families [5]. Third, SNP markers are dense enough that interference rules out double crossovers. In contrast, the previous study relied on estimation of double crossover rates using the Kosambi map function [15].

**Table 2:** Genetic map summary

	**Genetic length (cM)**		
**Chromosome**	**Male map**	**Female map**	**Sex averaged**
1	194.76	308.69	251.73
2	181.71	294.53	238.12
3	159.01	256.24	207.63
4	152.82	255.81	204.31
5	144.08	220.69	182.39
6	142.47	216.74	179.6
7	130.1	222.54	176.32
8	120.13	194.8	157.41
9	125.53.	187.45	156.49
10	126.25	205.56	165.91
11	115.17	179.01	147.09
12	121.78	193.35	157.56
13	106.86	138.4	122.63
14	92.64	134.42	113.53
15	103.85	134.49	119.17
16	108.93	155.9	132.42
17	107.32	144.89	126.1
18	95.44	130.97	113.21
19	92.53	107.51	100.02
20	96.41	108.97	102.69
21	48.13	64.93	56.53
22	62.5	77.58	70.04
x	-	175.35	-

Genetic map lengths for the Asian cohort. Map length is given by the average recombination rate among individuals on each chromosome, rather than the total number of crossovers divided by total meioses. We assume that our markers are dense enough to remove the possibility of undetected double crossovers.

**Table 3:** Summary of cohort results

	**Mongolian**	**Korean**	**CEPH families**
Number of SNP markers	569132	516452	931633
Meioses	254	722	234
Total crossovers detected	8776	24355	8093
Average male recombination rate	27.43	25.84	26.49
Average female recombination rate	41.96	40.75	42.90
Median resolution (bp)	83525	114681	74169
Historical hotspot concordance	0.83	0.81	0.82
Concordance of randomly placed intervals	0.36	0.37	0.35
Estimated true historical hotspot usage	0.74	0.70	0.72

Summary of results for the three cohorts. We do not find the differences in recombination rate or historical hotspot concordance significant. Historical hotspot concordance records the overlap rate of crossovers resolved to 30 kb or better. ‘Randomly placed intervals’ are generated by random perturbations of well-resolved crossovers following a normal distribution (μ = 0; σ = 200 kb). The Mongolian and Korean results were combined to form our Asian genetic map.

### Hotspot usage

**Figure 3:**
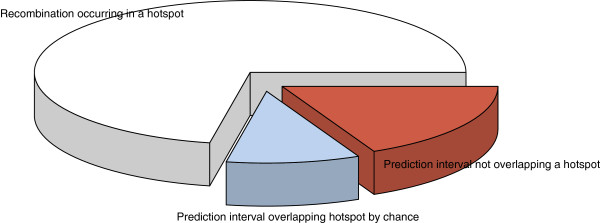
**Hotspot overlap of well-resolved crossovers.** Hotspot usage of Asian and CEPH families. Using recombination prediction intervals resolved to 30 kb or better, we counted the proportion that overlapped with known hotspots (white plus blue; 82%). To find a rough estimate for the proportion of these predictions where the recombination really occurred in a hotspot (white; 72%), we performed random perturbations. Our results show far more recombination occurring in historical hotspots than has been previously reported. Asian and CEPH families had almost identical hotspot usage.

We investigated the usage of the HapMap [9] set of hotspots by individuals. We call this the ‘historical hotspot’ concordance, because it represents divergence from recombination patterns established under a coalescent model. In addition to the hidden Markov model, our program also uses a deterministic scan to form a ‘prediction interval’ which defines the region in which a recombination event may have occurred. For this analysis, only 4,799 crossovers which could be pinpointed to within a prediction interval smaller than 30 kb were used, following the same criteria as previous studies [5]. We found that 82% of such intervals overlapped with a historical hotspot. Naturally, some of these overlaps were due to weak resolution, rather than because the recombination event actually happened in a hotspot. Discounting for this possibility by calculating the rate of random overlap (see Additional file 1) we estimate that about 72% of Asian recombination occurs in a historical hotspot. We searched for evidence of novel Asian hotspots using the set of non-concordant well-resolved crossovers, but only observed one cluster of more than two such crossovers. This was located on chromosome 18 from NCBI build 36 physical position 2,871,839 to 2,883,413. We repeated the above analysis for the CEPH families dataset. We found that 82% of well-resolved prediction intervals overlapped a hotspot in both Asians and CEPH families, discounted to 72% when taking overlaps occurring by chance into account (Figure 3). These measurements are in line with results from coalescent methods in which hotspots cover 71-79% of the genetic map [6]. Two family-based algorithms have previously been reported yielding lower hotspot overlap (72% for intervals resolved within 30 kb [5] and 74% for intervals resolved within 20 kb [18]) which we investigate further in Additional file 1.

### Maternal age effects

**Figure 4:**
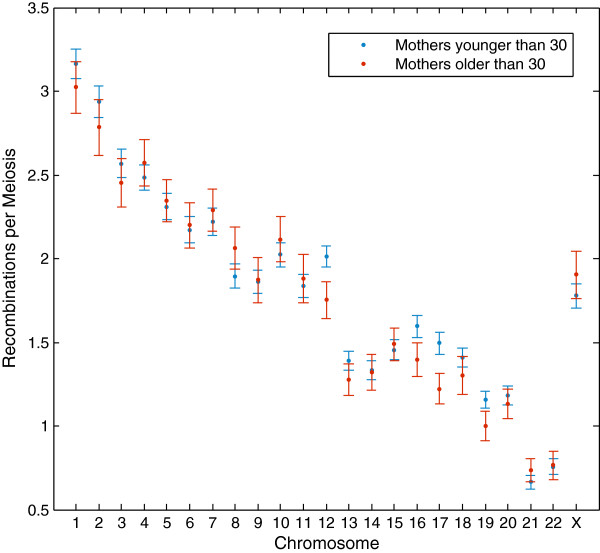
**Chromosome-specific recombination rate.** Comparison of the number of recombinations for women older than 30 (red) against those of age less than or equal to 30 (blue) at childbirth. Rates are shown for each of the chromosomes, including standard error. 328 children from the Mongol and Korean pedigrees are included in this dataset. Chromosomes 12 (p = 0.04) and 17 (p = 0.02) are significant if considered in isolation, but not after Bonferroni correction.

It is known that reduced recombination on chromosome 21 is associated with Down syndrome [19]. There is also a clear effect of maternal age on aneuploidy. It has thus been hypothesized that age-related aneuploidy could be caused by a reduction in recombination rates with maternal ageing. Early results, however, showed an opposite effect, with more crossovers detected in older mothers [5,20]. It was suggested that this was due to selection favouring oocytes with more crossovers protecting them against other deterioration in the meiotic system [20]. More recently, Hussin et al. [17] found a negative effect, and argued that more powerful effects on particular chromosomes (5 to 10, 15, 18 and 20) could reduce protection against non-disjunction. Our large sample size allowed us to address this problem by identifying the number of maternal recombinations in the meioses which gave rise to 338 children. This cohort was formed from all Asian families in our sample containing at least three children. Of these, 9 failed consistency checks and so were excluded from the analysis, and one was removed due to errors on the X chromosome. Recombination counts in these children were matched to their mother’s age at childbirth, rounded down to the nearest year. We performed regression analysis in R [21] with maternal ID included as a factor, so that we had a first-order model in maternal age at childbirth and maternal ID without interaction terms. We found a small unique effect of age on recombination rate of -0.29 recombinations for each year (p = 0.03; multiple R^2^ = 0.28; adjusted R^2^ = -0.003). At all ages, there is considerable variation in the recombination rate phenotype, and the linear model accounts for this variation poorly. We did not find a significant effect following a similar analysis for paternal age. We further tested for significant reductions on each chromosome between women younger and older than 30 (Figure 4). Chromosomes 12 and 17 showed a significant difference between the two age groups (p = 0.04 and p = 0.02; one-sided *T*-test), but this may be an artefact of multiple testing on 23 chromosomes. These chromosomes are also different to those identified as candidates previously [17]. This makes it difficult to argue that effects on particular chromosomes are responsible for age-related aneuploidy.

Given that the effects of age on recombination are small, they may be susceptible to discrepancies in methodology. In the study by Kong et al. [20], ages were rounded up to the nearest 5 years and only 1000 microsatellite markers of unknown position were used. Interestingly, Kong et al. found a slight decrease in recombination rate specifically between the ages of 25 and 30, and a higher proportion of the mothers in the Asian cohort fall within this age range. To investigate this, we sampled Asian mothers with an age distribution corresponding to the previous study and binned individuals into 5-year windows. However, these changes only resulted in a reduced effect of -0.23 recombinations per year. Our findings therefore do not fit with either the positive effects reported previously, or the much stronger and chromosome-specific negative effects reported more recently. The frequency of aneuploidy increases powerfully and exponentially with maternal age, whereas our regression analysis found a poor coefficient of determination and weak effect. Our results therefore contradict the hypothesis of a very simple, direct relationship between reduced recombination and increased aneuploidy with maternal ageing, although there may be more subtle links between the two.

## Conclusion

We have constructed the first high-resolution genetic map for Asians. Our map will be a useful resource for future population genetics research, improve the accuracy of linkage studies, help to predict disease-causing structural rearrangements and inform the design of genome-wide association studies in the Asian population. In general, Asian recombination patterns were similar to those in Europeans. A higher proportion of Asian and European recombination was mapped to hotspot regions than in previous studies. These results validate the application of combined genetic maps to Asians. Our data show that maternal age has a weak, negative effect on recombination rate, and there are no strong chromosome-specific effects.

## Methods

### Overview of methods

**Figure 5:**
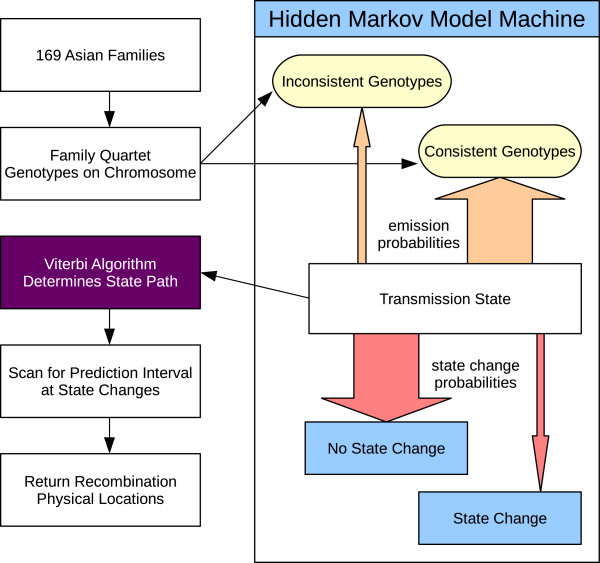
**Overview of methods.** An overview of our hidden Markov model’s application to Asian genotype data. Genotype data was gathered from SNP array assays of Korean and Mongolian family pedigrees. These pedigrees were split into family quartets and input into our algorithm. The hidden Markov model considers each SNP position as belonging to one of four transmission states. The transmission state at a position depends on the previous transmission state (pink arrows: the state is most likely to remain unchanged) and the family quartet’s genotypes (orange arrows: genotypes are expected to be consistent with the transmission state). The Viterbi algorithm determined the most likely sequence of transmission states of paternal and maternal alleles to the two children. At state changes, the algorithm scanned up and down to find the region where recombination may have occurred. The returned recombination prediction intervals were used to construct a genetic map, to analyse hotspot usage and to find the effect of maternal age on recombination rate.

We used nuclear family genotype data to find Asian recombination patterns. By directly observing recent recombination events, this approach avoids the problem of coalescent methods finding recombination receding many generations into the past. Several deterministic programs using parent-sibling tracing methods have been described in the literature [5,8,16,18], although few are available or easily applicable to new pedigree data. These methods have been shown to suffer from bias against calling recombination in smaller families [5]. Furthermore, they have demonstrated fairly low accuracy where validation by simulation was performed [16]. Therefore, we developed a new probabilistic method using a hidden Markov model. In general, our method was based on the idea that recombination events correspond to a parent switching between transmitting the same or different alleles to their children [22]. We classified combinations of family genotypes as consistent or inconsistent with these two modes of transmission, and used that classification to define a hidden Markov model (Figure 5). Given actual observed genotypes for families, we found the most likely sequence of transmission states and deduced that recombination occurred where states changed. Our algorithm is available as Python code (Additional file 4) for application to new datasets. The program is accurate, portable, quick and easy to use, and can handle standard PLINK format data.

### Algorithm design

At the core of our algorithm was a hidden Markov model which could find recombination events in family quartets consisting of a father, mother and two children. The input for this model was genotype data for SNPs on a single chromosome, encoded at each position as the alleles *A* and *B*. For any genomic locus in a parent, two possible modes of transmission are possible: both children may receive the same allele, or each child may receive a different allele. These paternal and maternal transmission states combine to yield four possible states. Where the transmission state differs at adjacent genomic loci, we can infer that a crossover occurred between them during a paternal or maternal meiosis. We model the sequence of states along the markers on a chromosome as a Markov process. Observed genotypes at a given locus in all four family members may be consistent with a subset of these states. For example, if the father, mother and child genotypes are *AA*, *AB*, *AA* and *AB* respectively at some SNP, then we can infer that the mother is transmitting different alleles to the two children, but can make no inference regarding paternal transmission. Homozygous loci are uninformative because they are consistent with all four states. A hidden Markov model was constructed with a state space consisting of the four transmission states, and transition weights between these states corresponding roughly to previously reported genome-wide recombination rates [6]. A hidden Markov model is an extension of a simple Markov chain, with observations also modelled with a probability distribution dependent on the state. This allows, for example, genotyping errors to be included in the model, rather than requiring post-hoc filtering. The set of emissions from each state was designed to encode all possible genotypes in all family members at a given locus, assuming a maximum of two alleles. The weights of emissions consistent with a transmission state were set to sum to 0.9995. Inconsistent emissions were allocated to the small remaining probability to allow for errors in DNA replication or genotyping. These small emission probabilities are balanced with the state transition probabilities to ensure that a few genotyping errors will not result in an erroneous double state transition. Family genotype data was fed into this model, and the Viterbi algorithm applied to find the most likely sequence of states. At each state change, an algorithm scanned forward and back to find the region of uncertainty, or ‘prediction interval’, where either neighbouring state was consistent with observations. For example, the Viterbi algorithm is able to determine a state change from both children inheriting the same allele from both parents, to the children inheriting different paternal alleles. However, the data will normally be ambiguous about the exact location of this change-point. To solve this problem, the set of possible states at each SNP in the region of the state change is recorded and a maximal interval formed by starting at the state change SNP and continuing in the 5^′^ and 3^′^ directions as far as more than one state remains possible. This non-probabilistic, strict delimitation of boundaries protects the algorithm from sensitivity to changes in state transition or emission probabilities. The SNPs marking the prediction interval were mapped to NCBI build 36 physical position. Alternative hidden Markov models were used to find maternal recombinations on the X chromosome (see Additional file 1). For families with an odd number of children, two overlapping quartets formed a quintet. Where a recombination prediction interval overlapped in the two quartets, we assume that the recombination occurred in the child present in both quartets and so only count it once. The collection of all prediction intervals was returned for further analysis.

### Validation

Our work uses a novel algorithm, although similar approaches have been applied to phase next-generation sequencing data [22,23]. We validated our algorithm by running it on simulated family data for which crossover locations were known. To simulate data as realistically as possible, we based our families on real haplotypes for chromosome 15 from the phased JPT+CHB HapMap dataset [9]. Parents were constructed by selecting two haplotypes each at random out of the 340 available. We then simulated four meioses, selecting 3 crossover locations at random in each, to generate two children. We constructed 1,000 family quartets in this way, containing 12,000 recombination events. Running our hidden Markov model on this data yielded 11,643 prediction intervals that correctly localised 97% of the synthetic crossovers. Out of these 11,643 prediction intervals, only one was incorrect. This means that the precision rate of our algorithm is greater than 99.99%. To check the robustness of our approach on real data, we compared our results to crossovers inferred from sparse microsatellite markers. We tested results in 6 family quartets on chromosome 19. This chromosome had 32 microsatellite markers typed in those family quartets, as well as in their grandparents, allowing discovery of crossovers using identity-by-descent. Out of 33 crossovers detected by our algorithm, 32 were consistent with the microsatellite data, and one corresponded to a 6 Mb distant crossover.

### Age effects

Where families contained more than two children, the children were split into mutually overlapping groups of three. Each such triple was then split into three overlapping pairs, which were input in turn together with parent genotype data into the hidden Markov model. The recombination rates in each child were derived from the rates in the overlapping quartets containing that child. This allowed consistency checks to identify results which contradicted with those of overlapping quartets. Before constructing our genetic map, we checked for such inconsistent triples, and filtered out all of the meioses implicated in these errors. There were four (partially overlapping) triples in three families where checks found mathematical inconsistency. We were able to rescue data for three children in one such family where they formed a consistent trio. This left 9 children in three families where recombinations could not be distinguished consistently. These were removed from the analysis.

### Data sources

This study used genotype data from Mongolian pedigrees, generated by the Illumina Human 610-Quad Beadchip yielding 569,132 markers. The data was originally collected for association studies in the GENDISCAN project. This was approved by the Institutional Review Boards of Seoul National University, approval number H-0307-105-002. Mendelian errors were filtered using PedCheck and double recombination errors were filtered using Merlin. Markers with a low call rate (<99%), high error rate (>1%) or low minor allele frequency (<0.01) were excluded as described previously [24]. We also used genotype data from the Healthy Twin Study, a project that genotyped adult twins and other family members willing to participate through two Korean hospitals. The Affymetrix Genome-Wide Human SNP Array 6.0 was used to type 906,600 SNPs overall, which was reduced to 516,452 after cleaning Mendelian and non-Mendelian errors. The criteria for exclusion were: Mendelian error in more than 3 families, minor allele frequency below 0.01, Hardy-Weinberg equilibrium test <0.001, missing genotype data >0.05 or non-Mendelian error in more than 3 families. The study protocol was approved by the ethics committees at Samsung Medical Center and Busan Paik Hospital. Results were compared to those obtained by applying the same method to CEPH pedigree data. This data was generated under the study entitled ‘Genotyping NIGMS CEPH Samples from the United States, Venezuela and France’, and provided to us under a dbGaP access request. There were 931,633 SNP markers in this dataset. From the complete pedigrees, families of at least four members were selected using a Python script. In all, 182 families were gathered in this way. The set of 32,991 (34,136 including the X chromosome) hotspots from HapMap Phase II [9] was used for historical hotspot concordance analysis after lifting to NCBI build 36. The software utility liftOver from UCSC (http://genome.ucsc.edu/cgi-bin/hgLiftOver) was used to perform this transfer, during which 6 hotspots were partially deleted.

## Competing interests

The authors declare that they have no competing interests.

## Authors’ contributions

JSS conceived of this study. JSS and JHS managed the GENDISCAN project and the Healthy Twin Study respectively. TB constructed the hidden Markov model, analysed the data and drafted the manuscript. YSJ helped the analysis. All authors participated in manuscript writing. All authors read and approved the final manuscript.

## Supplementary Material

Additional file 1 **Supplementary Notes.** Details on X chromosome hidden Markov models, concordance analysis, alternative simulations, algorithm comparison and Mongolian exome sequencing results. Click here for file

Additional file 2 **List of Crossover Prediction Intervals.** The complete unordered list of recombination events detected in Mongolian and Korean families. The first column is the sex of the individual in which recombination was observed, the second column is the chromosome on which it was located, and the third and fourth mark the physical position of the start and end point of the range in which the algorithm determined the recombination could have occurred. Positions are given in terms of NCBI build 36. Click here for file

Additional file 3 **Genetic Maps. Archive containing combined genetic maps for all chromosomes.** The SNP list text files contain the rs numbers of all of the SNPs shared by the Mongolian and Korean cohorts, ordered by physical position. The map files give the genetic distance between each of these markers in order in centimorgans. All observed Asian recombination events are mixed to form this map. Click here for file

Additional file 4 ** Quartet Analysis Algorithm. Archive containing a script to be opened using Python (http:////python.org).** The Python source code for the algorithm used in this study (script.py) is available to run on new datasets. Given the identities of the father, mother and two children in a family quartet, and genotype data in PLINK format, it returns a list of recombination prediction intervals. For instructions and input file requirements, please see the file README.txt. This package also includes example input files (*.syn) generated during simulations. Click here for file
