# Supplementary material for: Fine-scale mapping of meiotic recombination in Asians
Source: BMC Genet. 2013 Mar 8;14:19. doi: 10.1186/1471-2156-14-19 (PMC3599818; doi:10.1186/1471-2156-14-19)
Supplement: Additional file 1 — Supplementary Notes. Details on X chromosome hidden Markov models, concordance analysis, alternative simulations, algorithm comparison and Mongolian exome sequencing results. [file 1471-2156-14-19-S1.pdf]

# Fine-scale mapping of meiotic recombination in Asians

## Supplementary notes

X chromosome hidden Markov models

Concordance analysis

Alternative simulations

Algorithm comparison

Mongolian exome sequencing results

Supplementary references

# X chromosome hidden Markov models

In the regions on the X chromosome which are not pseudo-autosomal, the father only carries a single allele and paternal recombination does not occur. The four transmission states used in the standard autosomal hidden Markov model are not appropriate for these regions. We constructed alternative hidden Markov models to find transmission changes in quartets on the X chromosome. This was complicated by differences between emission consistencies for quartets with two daughters, two sons and one child of each sex. To take this into account, we constructed a different HMM for each of those three cases, with only two transmission states depending on whether the mother gave the same allele to both children or different alleles. As with the autosomal method, the four individuals' genotypes were converted into a format with just two alleles. Those quartet genotypes that were consistent with a state were given emission probabilities summing to 0.995, and the inconsistent genotypes allocated the remainder. Values were converted to log probabilities and then the genotype observations were fed into the Viterbi algorithm. Only maternal recombinations were detected and reported in this way.

After the application of our algorithm, we found that one mother had 133 crossovers on chromosome X in the meiosis leading to one of her children. This is obviously incorrect, and is the only such outlier that we found. This must have been due to a bug in our program, or perhaps incorrect input data. In any case, we removed these erroneous crossovers from analysis - they are not included in the genetic map for the X chromosome, the mother was removed from calculations of global map length, and the child was not included in the maternal age linear regression.

# Concordance analysis

From the set of all recombination prediction intervals, only those resolved to an accuracy of 30 kb or better were used for analysis of concordance with historical hotspots. A python script was used to find the proportion of each parent's set of recombinations which overlapped with a historical hotspot. The average concordance for individuals in CEPH was 82%, the concordance for Mongolians was 83% and concordance for Koreans was 81%. There was considerable variation amongst individuals in their concordance rate

phenotype. It should be noted that the average number of recombination events on which this calculation was performed was much larger in CEPH, which accounts for reduced variance in this sample. We also calculated the number of prediction intervals which overlapped with hotspots with different resolution thresholds. After combining all events among Asians, we found 84% of intervals under 40 kb, 87% of intervals under 60 kb and 90% of intervals under 100 kb overlapped with hotspots. We also calculated the proportion of intervals overlapping with an alternative set of hotspots provided by the pilot to the 1000 Genomes Project. There were 32,045 hotspots in this collection, which were restricted to the autosomes. We found that 79% of Asian autosomal recombination prediction intervals under 30 kb overlapped with one of these hotspots.

A proportion of the recombination prediction intervals overlapped with historical hotspots due to weak resolution, rather than because the recombination event actually happened in a hotspot. In order to investigate the true hotspot usage, we first assume that all crossover events which happen in a hotspot are correctly mapped to that locus. Then the total number of observed intervals not overlapping with a hotspot must be given by the number of crossovers genuinely not occurring in a hotspot, multiplied by the probability that their interval of uncertainty does not overlap a hotspot by chance. The latter probability was derived by genome-wide simulations using the output prediction intervals from three populations to allow for differences in local SNP densities and lengths of uncertainty intervals. To ensure that medium-scale rate constraint was maintained, simulation proceeded by perturbation of the observed prediction intervals. We ignore the very small chance that this perturbation might move a crossover so that it again occurred in another hotspot. As with the analysis of concordance, only prediction intervals resolved to within 30 kb were used. Each interval was moved by a random distance (normally distributed with mean 0 and standard deviation 200 kb), and its new position was tested for overlap with hotspots. This is essentially an arbitrary cut-off to protect medium-scale rate constraint. As current understanding of such regulation is poor, other studies have also used arbitrary perturbation limits (we use the same values as Coop et al.[1] to make comparison between results more direct). Simulation of 100 iterations yielded a 0.3504 concordance rate for randomly placed intervals in CEPH, and 0.3645 for combined Mongol and Korean intervals. The proportion of crossovers that did not occur in a hotspot is given by the proportion of well-resolved prediction intervals that did not overlap a hotspot, divided by 1 minus the proportion of well-resolved crossovers not in hotspots themselves but whose prediction interval overlaps a crossover. This implies that only 28% (0.18 / (1-0.3645))

of Asian recombination occurs outside of a hotspot.

Both the basic proportion of overlaps and also the discounted hotspot usage estimate we derived were considerably higher than numbers in previous papers. To check if this was an artefact of our concordance calculation procedures, we performed the same analysis using the set of crossover intervals supplied by Fledel-Alon et al.[2] as supplementary material to their manuscript. We found that only 63% of the well-resolved crossovers in this dataset overlapped with a HapMap hotspot. Since our analysis also included the X chromosome, we investigated if this could cause bias. However, repeating the calculations with X chromosome prediction intervals excluded had little effect (for example Asian overlap was 82% without X).

We gathered the set of all prediction intervals from the Asian pedigrees which did not cover a historical hotspot and were resolved to within 30 kb. We looked for genomic regions with clusters of such intervals as potential evidence for novel Asian hotspots. From this prediction interval set, a triple overlap was only observed in one region, from physical position 2,871839 to 2,883,413 on chromosome 18.

# Alternative simulations

We performed simulation of meiosis events and ran our hidden Markov model on the pedigree files generated. The simulation procedure first selected positions in the genome at random as sites of recombination, then child haplotypes were constructed following the parental haplotypes as templates. For each child's paternally inherited haplotype, one of the two haplotypes in the father was selected at random to begin with, and the child's chromosome generated from this. When a recombination point was reached, the paternal haplotype was switched and SNP alleles were then taken from the other. Generation of maternally inherited haplotypes proceeded similarly. Genotypes were sorted before writing to file, so the hidden Markov model was blind to which haplotype was which. Each round of simulation generated four files: a family list, giving the codes for the father and mother in terms of their combined HapMap haplotype codes, and the codes 'child1' and 'child2' for the children; an ordered list of SNP physical positions taken from the HapMap data files; a pedigree file containing the genotypes of the four family members encoded in PLINK format; a list of the randomly selected physical positions at which the paternal and maternal crossovers occurred. The

first three of these files were input directly into the algorithm (Additional File 4) while it was blind to the contents of the fourth file listing true recombination locations. We then ran code to compare the output of the hidden Markov model with the true crossover position list. To calculate sensitivity, we counted the number of paternal recombination events which lay within an output paternal prediction interval and likewise for maternal events. We define the precision rate as the proportion of prediction intervals which in fact contained a true recombination event (from the right parent). Results of this validation for 1000 iterations on chromosome 15 are given in the main manuscript. In addition to the results for chromosome 15, we also tested other chromosomes and other values for the number of crossovers per meiosis. In these simulations, 100 different family pedigrees were generated for each set of parameters. Parameters were designed to test a variety of different chromosomes with various numbers of crossovers per meiosis. Results are given in the table below.

| Chromosome | Crossovers per Meiosis | Sensitivity | Precision Rate |
|---|---|---|---|
| 15 | 3 | 97.03% | 99.99% |
| 1 | 4 | 98.25% | 100% |
| 5 | 4 | 98.25% | 100% |
| 21 | 2 | 96.5% | 99.87% |
| 7 | 1 | 99.25% | 100% |

We also tested the algorithm on a dataset generated with 25 crossovers per meiosis on chromosome 16.

| Chromosome | Crossovers per Meiosis | Sensitivity | Precision Rate |
|---|---|---|---|
| 16 | 25 | 81% | 99.49% |

Sensitivity was very poor this biologically unlikely scenario. This may be due to transition probabilities being optimised for more plausible recombination rates, such that close double recombinations were interpreted as genotyping errors by the model. In general, this validation shows greater accuracy than has been previously demonstrated for other similar programs. We compare these results with those of Ting et al. [3] where simulations revealed a sensitivity of 89% and where 92% of prediction intervals contained simulated crossovers.

# Algorithm comparison

A number of different algorithms have been developed to locate recombination events given family genotypes. Some use probabilistic approaches similar to the hidden Markov model we introduced. For example, Roach et al. used the idea of switching between identical and non-identical inheritance of alleles to phase nuclear families using whole-genome sequencing data[4]. A hidden Markov model was also described in the supplementary materials to the work by Fledel-Alon et al.[2]. In this application, Markov Chain Monte Carlo methods were used to obtain estimates for parental haplotypes based on whole family genotype data. Given this prior information, a hidden Markov model could then find haplotype switches in children. Several other algorithms used non-probabilistic approaches based on phasing families and then tracing alleles from parents to children[1, 3]. One deterministic program, called PSTReader, was made available as MATLAB code by its authors[5].

In order to compare our algorithm with the previous parent-sibling tracing approach, here we present a comparison of the output of PSTReader with that of our algorithm on chromosome 1 for paternal recombination in five family quartets. We formatted genotype data on chromosome 1 for each individual, then input together with SNP position information. PSTReader output a figure showing a sequence of states, which we label arbitrarily 0 and 1. Similarly to our hidden Markov model, PSTReader works by capturing state changes which indicate crossover events. The regions where PSTReader is transitioning between the two states thus correspond to predicted paternal recombinations. Most such transitions appear as vertical lines below because prediction intervals are small compared to genome size. We ran our own algorithm on the same five family quartets and added the output paternal recombination predictions to the figures below.

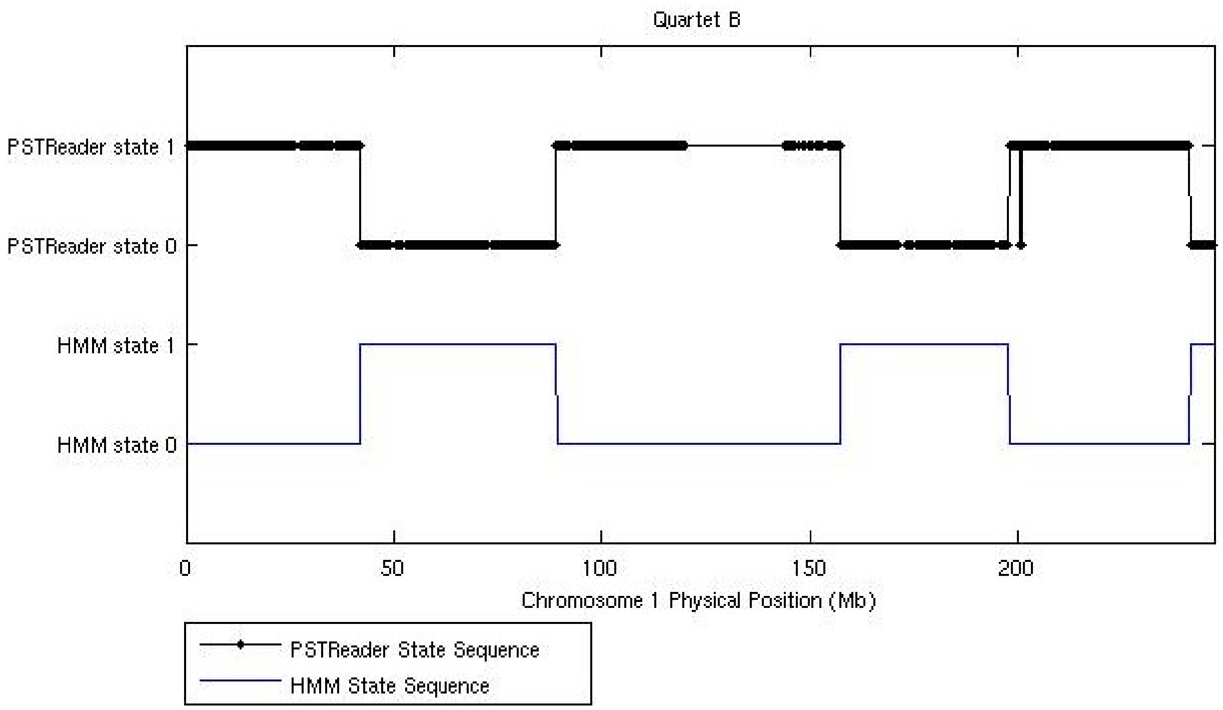
Quartet B
PSTReader state 1
PSTReader state 0
HMM state 1
HMM state 0
0
50
100
150
200
Chromosome 1 Physical Position (Mb)
PSTReader State Sequence
HMM State Sequence

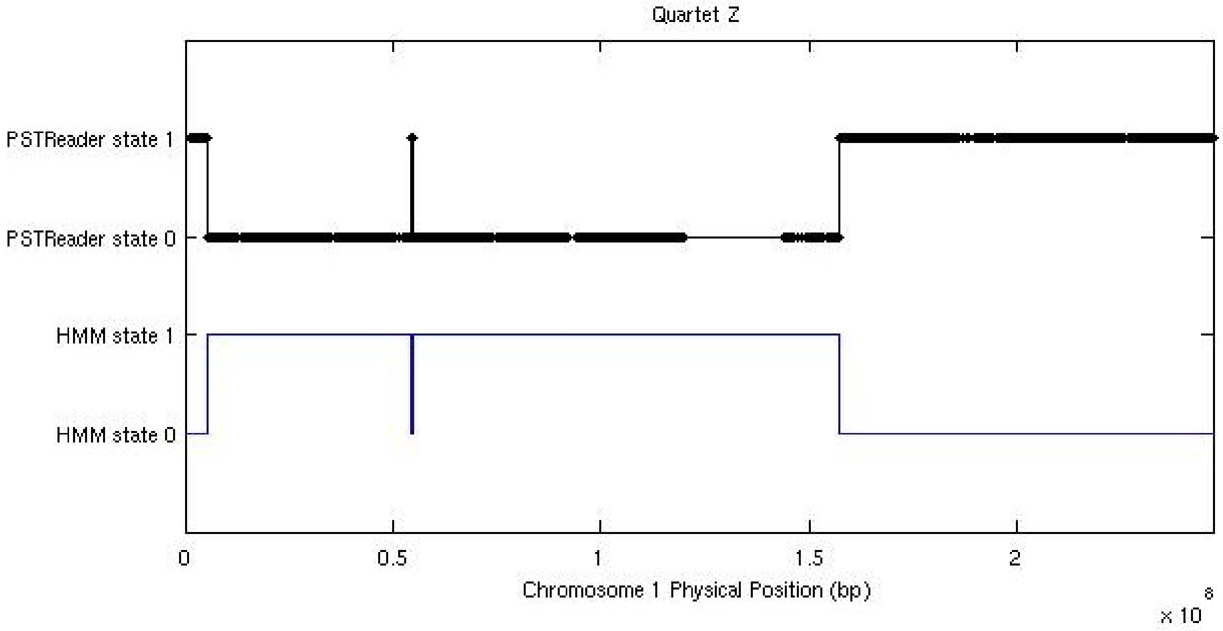
Quartet Z
PSTReader state 1
PSTReader state 0
HMM state 1
HMM state 0
0
0.5
1
1.5
2
Chromosome 1 Physical Position (bp)
x 10^8

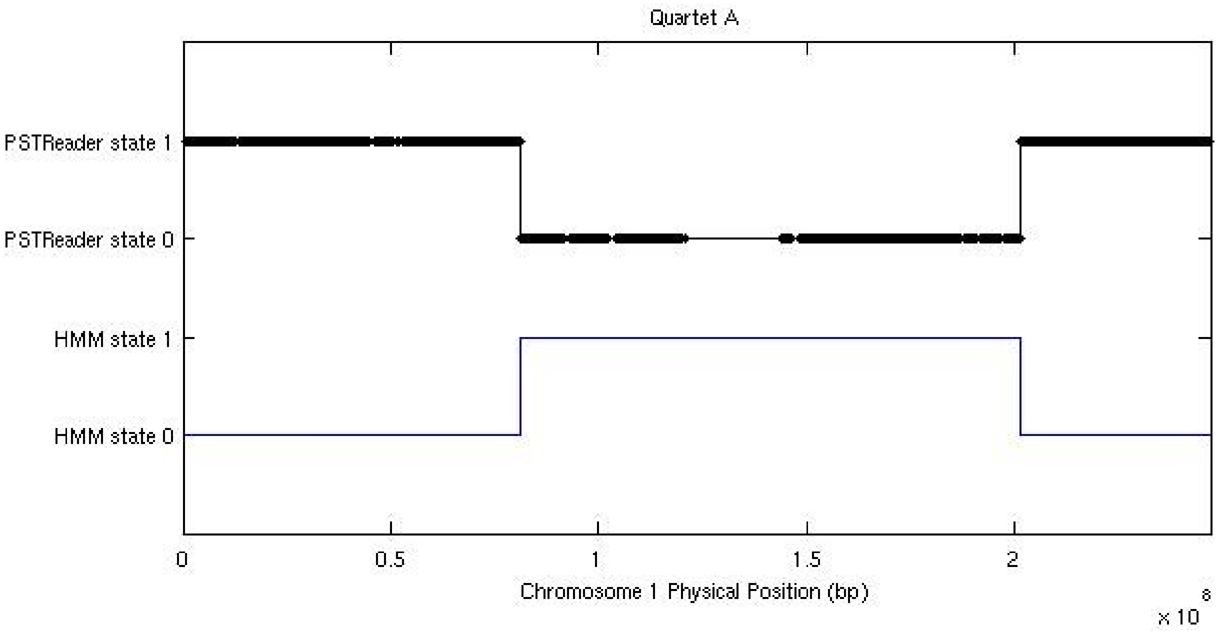

Quartet A
PSTReader state 1
PSTReader state 0
HMM state 1
HMM state 0
0
0.5
1
1.5
2
Chromosome 1 Physical Position (bp)
x 10^8


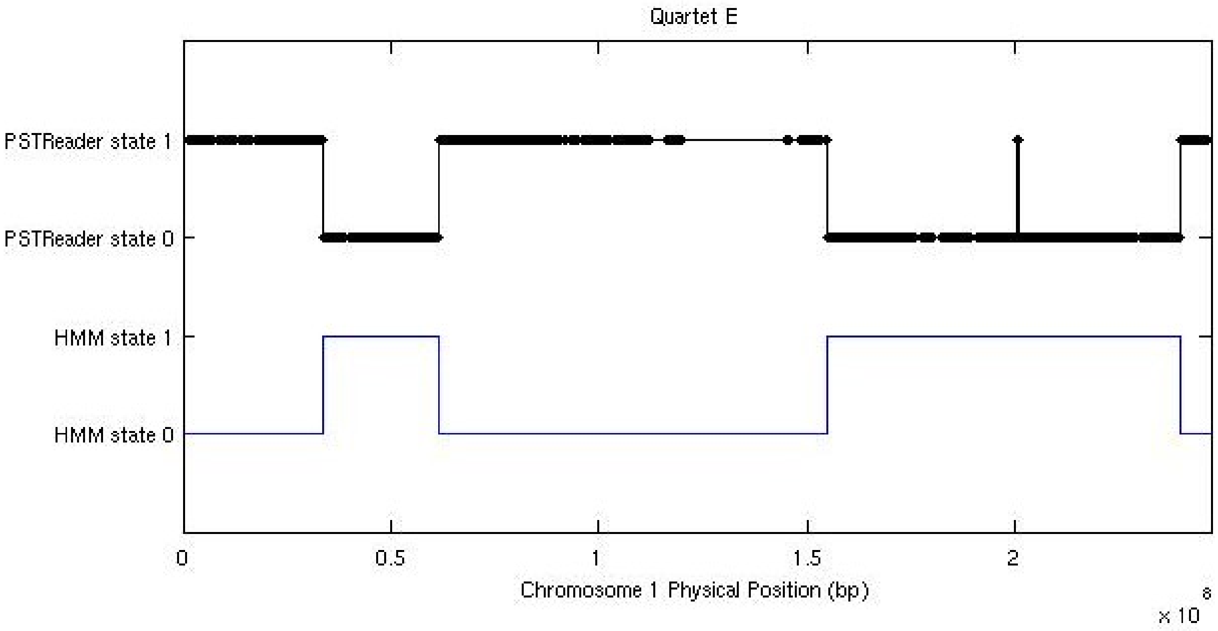

Quartet E
PSTReader state 1
PSTReader state 0
HMM state 1
HMM state 0
0
0.5
1
1.5
2
Chromosome 1 Physical Position (bp)
x 10^8

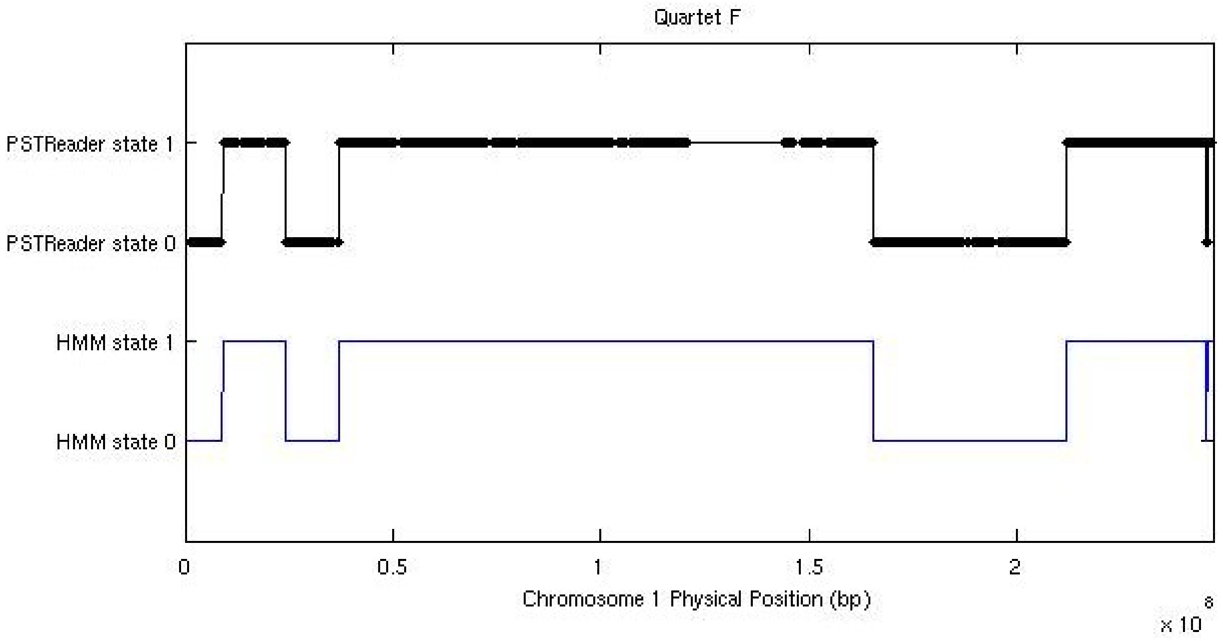


Generally, the two algorithms are in very good agreement. All 22 crossovers predicted by the hidden Markov model are also predicted by PSTReader. However, PSTReader also predicts four crossovers that the hidden Markov model does not. These are both close double-recombinations (quartets B and E, physical position ~200 Mb). Although there is no gold-standard against which to check, they appear to be errors caused by incorrect genotyping. Interestingly, these two double-crossovers occur in the same part of chromosome 1, which suggests that a particularly error-prone region or single SNP could be responsible for repeated errors.

Deterministic parent-sibling tracing algorithms use post-hoc filtering of results to handle genotype errors, rather than including them in a statistical model. To investigate our handling of genotype errors, we changed our model by setting the parameter for the proportion of expected genotype errors to a much lower value of $5x10^{-9}$ (this value was originally 0.0005), corresponding more closely with a deterministic approach. Running this altered algorithm on the Mongolian family data, we counted a total of 8,867 prediction intervals. This is notably higher than the 8,776 predictions by the original model. The hotspot overlap of well-resolved (<30 kb) prediction intervals from the altered model was 82%.

In our analysis of historical hotspot concordance, we found that 82% of well-resolved (ie. shorter that 30kb) hidden Markov model recombination prediction intervals overlapped a hotspot in both Asians and CEPH

families, discounted to 72% taking overlaps occurring by chance into account. Two previous algorithms have been reported yielding lower hotspot concordance. Firstly, Coop et al. found 72% of well-resolved predictions overlapping hotspots, discounted to 60% taking random overlaps into account[1]. Using a different resolution threshold of 20kb, Khil and Camerini-Otero found 74% of intervals overlapping, again in a European population[6]. Because concordance rates for CEPH families in our study are higher (~10 percentage points) than for Europeans segregating unexceptional *PRDM9* alleles (including the Framingham Heart Cohort and Autism Genetic Resource Exchange) in other studies[1, 2], it is likely that the disparity is due to methodology, rather than real ethnic difference. To investigate further, we compared our algorithm with NFTOOLS[7], a derivative of the original algorithm by Coop et al.[1]. Using NFTOOLS, we first confirmed its accuracy using simulated data as previously described in Methods. Secondly, we applied it to our Mongolian dataset using standard parameters.

(1) On simulated data, NFTOOLS yielded a precision rate of 100% and sensitivity of 98% (based on 100 iterations). These results are very similar to our own algorithm, supporting the high accuracy of both programs. (2) When applied to Mongolian family data, 94% of the 8,969 output recombination prediction intervals overlapped with a prediction by our hidden Markov model. In the Venn diagram below, only predictions for the autosomes are shown, since NFTOOLS does not work on the X chromosome. We consider predictions shared if any part of their prediction intervals overlap.

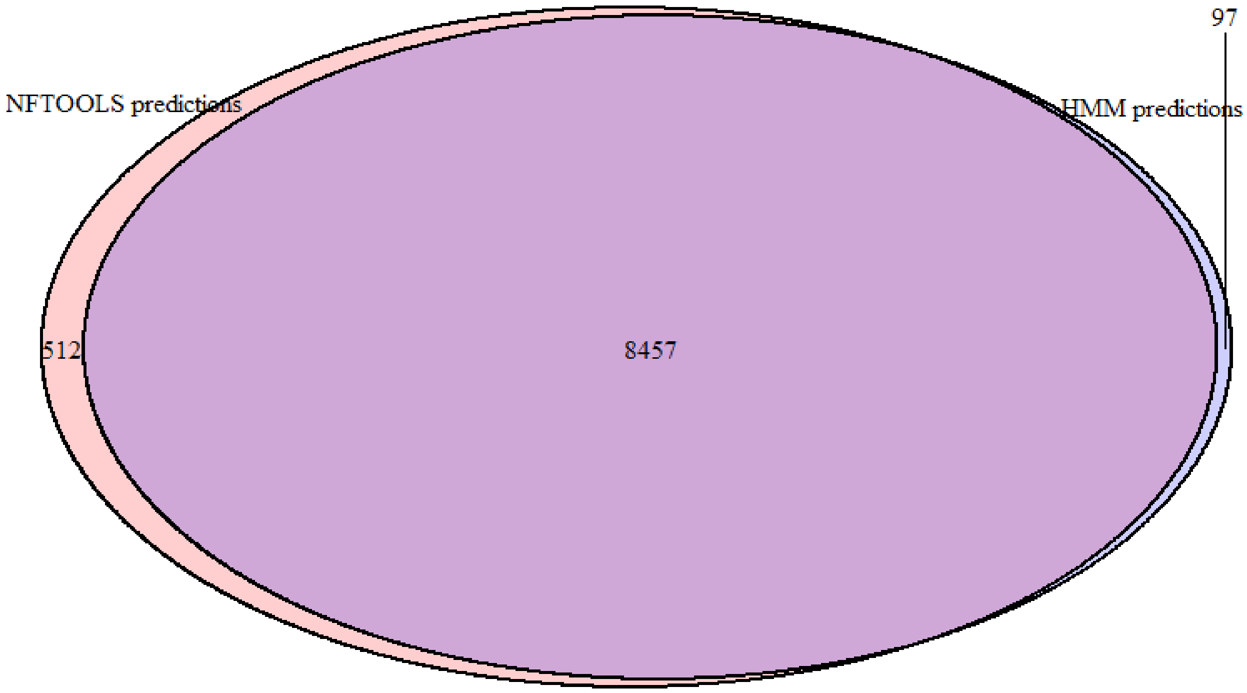


The median length of NFTOOLS prediction intervals was 64,514 bp, compared to the median for our hidden Markov model of 83,525 bp. In general, our algorithm uses looser criteria to generate more conservative prediction intervals than NFTOOLS. Rather than attempting to reduce the interval to the closest informative markers, we include the entire region of uncertainty around a state switch as described in Methods. This difference in prediction interval delimitation may contribute to the different hotspot overlap rates observed, since a set of intervals with larger lengths will tend to have greater overlap, either by chance or by fewer errors due to more conservative criteria. In particular, the mean length of well-resolved (<30 kb) NFTOOLS prediction intervals was 14,013 bp compared to 17,221 bp for our hidden Markov model. The historical hotspot overlap of the 2,806 well-resolved NFTOOLS prediction intervals was 76%, which is higher than previously reported[1, 7]. We performed random perturbations as described previously and estimated a random overlap rate of 31%. Using this to attempt to discount against the effects of different interval sizes in overlap rates, we estimate that 65% of recombination predictions occurred in a historical hotspot. Interestingly, of the 679 well-resolved NFTOOLS recombination prediction intervals which did not overlap a hotspot, 48% were matched with a prediction by the hidden Markov model which itself was overlapping a

hotspot, although only 45 of these 325 matched predictions were themselves well-resolved. Finally we investigated whether the NFTOOLS predictions contained more close double recombinations by searching for the nearest crossover to each prediction within the same parent. Strikingly, the median distance to the nearest other crossover for NFTOOLS predictions that concurred with a hidden Markov model prediction (n=8,457) was 11,003,674 bp, while the median for those predictions that were unique to NFTOOLS (n=512) was 42,302 bp. This observation is similar to that for PSTReader above, which may be interpreted as greater sensitivity to rare close double recombinations or to genotyping errors. Predictions corresponding to genotype errors are likely to decrease hotspot overlap rates due to the low (~30%) overlap of randomly placed intervals. Combining (1) and (2), we can conclude that both NFTOOLS and our hidden Markov model are accurate in crossover detection, and much of the discrepancy in hotspot usage may result from different thresholds for prediction intervals. A large proportion of the crossovers counted as not overlapping a hotspot by NFTOOLS would be counted as overlapping if a more conservative prediction interval was used. In addition, we can expect that other differences between studies, such as marker differences, genotyping error handling, and size and structure of enrolled families may contribute to the discrepancy.

# Mongolian exome sequencing results

Our research group performed a survey of exonic variants within the Mongol population by exome sequencing. The exomes of 38 unrelated Mongolian individuals were sequenced using DNA extracted from blood. Genomic DNA was sheared and used for the construction of a paired-end sequencing library following the standard Illumina protocol. The SureSelect Human All Exon 50Mb Kit (Agilent Inc.) was used to enrich for exonic sequences, and sequencing was performed on a Hiseq2000 sequencer. This yielded an average of 36 million 100 bp reads for each individual. After successful alignment of 32 million reads by GSNAP, we used filter conditions to identify genomic variants. We investigated the mutations of PRDM9 in this dataset by selecting only those variants within its exons as defined by RefSeq. A total of 6 different SNPs were found at low frequencies. The most common of these was heterozygous in 16 out of 38 individuals. All of these SNPs caused changes to the protein sequence.

| RS# | HG19 position | Frequency | Residue position relative to alpha helix start | Effect |
|---|---|---|---|---|
| rs77287813 | 23527565 | 5% | 3 | N>H |
| rs6875787 | 23527239 | 21% | 6 | T>S |
| rs61051796 | 23527637 | 4% | 3 | S>R |
| rs112815500 | 23527492 | 1% | 6 | R>S |
| novel | 23527323 | 3% | 6 | |

The residues of a C2H2 zinc finger predicted to affect DNA binding motif lie at positions -1, 3, 6 relative to the start of the alpha helix[8]. Surprisingly, all of the SNPs that we observed were in amino-acids at those positions. This reflects the powerful drive to generate new hotspots under a Red Queen dynamic. We found two novel adjacent SNPs, both present in two individuals. Such mutations likely represent a source of deviation in recombination patterns within the Mongolian population.

# Supplementary references


1. Coop G, Wen X, Ober C, Pritchard JK, Przeworski M: **High-Resolution Mapping of Crossovers Reveals Extensive Variation in Fine-Scale Recombination Patterns Among Humans**. *Science* 2008, **319**:1395–1398.

2. Fledel-Alon A, Leffler EM, Guan Y, Stephens M, Coop G, Przeworski M: **Variation in Human Recombination Rates and Its Genetic Determinants**. *PLoS ONE* 2011, **6**:e20321+.

3. Ting JC, Roberson ED, Currier DG, Pevsner J: **Locations and patterns of meiotic recombination in two-generation pedigrees.** *BMC medical genetics* 2009, **10**:93+.

4. Roach JC, Glusman G, Smit AFA, Huff CD, Hubley R, Shannon PT, Rowen L, Pant KP, Goodman N, Bamshad M, Shendure J, Drmanac R, Jorde LB, Hood L, Galas DJ: **Analysis of Genetic Inheritance in a Family Quartet by Whole-Genome Sequencing**. *Science* 2010, **328**:636–639.

5. Lee Y-SS, Chao A, Chen C-HH, Chou T, Wang S-YMY, Wang T-HH: **Analysis of human meiotic recombination events with a parent-sibling tracing approach.** *BMC genomics* 2011, **12**.

6. Khil PP, Camerini-Otero RD: **Genetic Crossovers Are Predicted Accurately by the Computed Human Recombination Map**. *PLoS Genet* 2010, **6**:e1000831+.

7. Hussin J, Roy-Gagnon M-H, Gendron R, Andelfinger G, Awadalla P: **Age-Dependent Recombination Rates in Human Pedigrees**. *PLoS Genet* 2011, **7**:e1002251+.

8. Pabo CO, Peisach E, Grant RA: **Design and selection of novel Cys2His2 zinc finger proteins.** *Annual review of biochemistry* 2001, **70**:313–340.
